# Combination of *Lactobacillus acidophilus* and *Bifidobacterium animalis* subsp. *lactis* Shows a Stronger Anti-Inflammatory Effect than Individual Strains in HT-29 Cells

**DOI:** 10.3390/nu11050969

**Published:** 2019-04-27

**Authors:** Sing-Chung Li, Wei-Fang Hsu, Jung-Su Chang, Chun-Kuang Shih

**Affiliations:** 1 School of Nutrition and Health Sciences, College of Nutrition, Taipei Medical University, Taipei 11031, Taiwan; sinchung@tmu.edu.tw (S.-C.L.); greg_hsu@hotmail.com (W.-F.H.); susanchang@tmu.edu.tw (J.-S.C.); 2 Graduate Institute of Metabolism and Obesity Sciences, College of Nutrition, Taipei Medical University, Taipei 11031, Taiwan; 3 School of Food Safety, College of Nutrition, Taipei Medical University, Taipei 11031, Taiwan; 4 Master Program in Food Safety, College of Nutrition, Taipei Medical University, Taipei 11031, Taiwan

**Keywords:** intestinal epithelial cells, inflammation, probiotics, *Lactobacillus acidophilus*, *Bifidobacterium animalis* subsp. *lactis*

## Abstract

Inflammatory bowel disease (IBD) is an emerging health problem associated with the dysregulation of the intestinal immune system and microbiome. Probiotics are able to reduce inflammatory responses in intestinal epithelial cells (IECs). However, entire signaling pathways and the interaction between different probiotics have not been well-documented. This study was designed to investigate the anti-inflammatory effects and mechanisms of single and combined probiotics. HT-29 cells were induced by lipopolysaccharide (LPS) and tumor necrosis factor (TNF)-α, treated with *Lactobacillus acidophilus*, *Bifidobacterium animalis* subsp. *lactis* or their combination and analyzed for inflammation-related molecules. Both *L. acidophilus* and *B. animalis* subsp. *lactis* reduced interleukin (IL)-8 secretion and the expressions of phosphorylated p65 nuclear factor-kappa B (*p*-p65 NF-κB), phosphorylated p38 mitogen-activated protein kinase (*p*-p38 MAPK), vascular cell adhesion molecule-1 (VCAM-1) and cyclooxygenase-2 (COX-2), while they increased toll-like receptor 2 (TLR2) expression. *L. acidophilus* did not decrease intercellular adhesion molecule-1 (ICAM-1) but enhanced the inhibitory efficacy of *B. animalis* subsp. *lactis*. Combined probiotics showed the best anti-inflammatory activity. These results suggest that *L. acidophilus* and *B. animalis* subsp. *lactis* may exert a potent anti-inflammatory effect through modulating TLR2-mediated NF-κB and MAPK signaling pathways in inflammatory IECs. Both strains, especially their combination, may be novel adjuvants for IBD therapy.

## 1. Introduction

Inflammatory bowel disease (IBD), including ulcerative colitis (UC) and Crohn’s disease (CD), is traditionally a common chronic gastrointestinal disease in several Western countries. The highest reported prevalence rates of IBD are in Europe (505/100,000 for UC in Norway and 322/100,000 for CD in Germany) and North America (286/100,000 for UC in the USA and 319/100,000 for CD in Canada) [1]. The overall prevalence of IBD has exceeded 0.3% in North America and many European countries [1]. However, IBD has become a global disease with accelerating incidence in newly industrialized countries in Asia, Africa and South America [1]. According to a recent study of Yen et al. [2], both the incidence and prevalence of IBD increased continuously in Taiwan from 2001 to 2015. Improved hygiene, civilization and Westernization may be possible causes [2]. Several studies suggest that IBD is an emerging health problem in the world [1,2,3]. The reason for the global increase in this initially Western-prevalent disease is still unknown, but it may be closely associated with environmental and social changes, such as the adoption of Western lifestyle and diet [1,2]. The rise of IBD in adults and children has been observed in Western developed countries, while gut microbiota and dietary fiber-rich diet of rural African individuals may protect them from the threat of IBD [4].

The etiology of IBD is thought to relate to a complex interaction of genetic and environmental factors, including the dysregulation of the enteric immune system and alterations in the intestinal microbiome [5]. Patients with IBD have typical dysbiosis characterized by an increase in pro-inflammatory bacteria, a decrease in anti-inflammatory bacteria and a reduction in bacterial diversity compared to the healthy population [5]. A reduced diversity of the gut microbiota in IBD patients is largely due to a low abundance of probiotics belonging to the *Lactobacillus* and *Bifidobacterium* genera [6]. IBD is characterized by mucosal and systemic inflammatory responses to the gut microbiome, which is the outcome of a lack of mucosal immune tolerance [6]. It is well-known that chronic inflammation is related to carcinogenesis and thus long-standing IBD is considered as a risk factor of colorectal cancer (CRC), especially colitis-associated colorectal cancer (CAC). In recent years, high incidence rates of CRC have been observed in Taiwan and other countries where the rates were historically low [7]. This increasing trend of CRC is similar to that of IBD in many countries. Both CRC and IBD are now global health issues that cannot be ignored.

The mucosal tissue of the intestine contains the largest part of the immune system in humans. It is estimated that 70% of immune cells are present in the gut [8]. Intestinal epithelial cells (IECs) provide a physical and chemical barrier between the intestinal lumen and the lamina propria [8]. They represent the first contact point for bacteria within the gut and thus prevent microbial penetration and induce communication for immune recognition of intestinal bacteria [9]. The activation of the pro-inflammatory genes in IECs in response to challenges by bacterial products such as lipopolysaccharide (LPS) or pro-inflammatory cytokines such as tumor necrosis factor (TNF)-α is associated with acute and chronic intestinal inflammation [10]. However, bacterial ligands may not directly alter the inflammation of IECs, although bacterial cell wall components, such as LPS and lipoteichoic acid (LTA), contribute to toll-like receptor (TLR)-mediated inflammation [11]. Ligands from pathogenic bacteria efficiently activate monocytes and macrophages through the secretion of pro-inflammatory cytokines such as TNF-α, interleukin (IL)-1β, IL-6 and IL-8 [11]. In contrast, ligands from probiotic bacteria only minimally induce TNF-α production [11]. These studies suggest that it is reasonable to simulate the inflammatory environment of the intestine using a combination of bacterial ligands and pro-inflammatory cytokines.

Probiotics have beneficial effects on the host through their ability to modulate the mucosal immune system. They have been shown to both augment/modulate homeostatic immune defenses and to ameliorate infection, inflammation and allergy by modulating gut function [12]. Treatment with probiotics has been considered to be potentially effective and safe in patients with IBD. It has been reported that individual probiotic species have variable potential to stimulate the mucosal immune system [13]. Probiotics differentially modulate IECs’ responses via the activation or suppression of distinct signaling pathways, such as TLR, nuclear factor-kappa B (NF-κB) and mitogen-activated protein kinase (MAPK) signaling pathways, in a strain-dependent manner, including strains of the same species [12,14].

The majority of probiotic microorganisms belong to the genera *Lactobacillus* and *Bifidobacterium*, and most *Lactobacillus* and *Bifidobacterium* species belong to probiotics [15]. *Lactobacillus* and *Bifidobacterium* species have been shown to reduce inflammatory responses, including NF-κB activation and IL-8 production, in inflammatory IECs, rodent colitis models and patients with IBD [9,16]. However, not all probiotics exert constant anti-inflammatory effects in experimental models of intestinal inflammation. For example, *L. acidophilus*, *B. lactis* and *L. casei* have an anti-inflammatory activity in the 2,4,6-trinitrobenzene sulfonic acid (TNBS) model of rat colitis but each probiotic shows its own anti-inflammatory profile [17]. It would be interesting to compare different probiotics in the same experimental model, in order to establish the best profile in a given setting and to further apply this new concept for IBD therapy [17]. A recent meta-analysis study demonstrated that results from clinical trials of probiotics on IBD were inconsistent [5]. Among all trials included in this meta-analysis, some showed an improvement in the maintenance or induction of remission by probiotics, while others failed to show any benefit [5]. This could be due to the species or strains of probiotics used, or the methodological differences among studies. Some studies found that probiotics may be as effective as 5-aminosalicylates (5-ASAs), a common drug for the treatment of IBD, in preventing relapse of quiescent UC but other studies showed that there was no benefit of probiotics over placebo in inducing remission of active UC [5]. However, when only trials of VSL#3—a mixed preparation of probiotics—were considered, there appeared to be a benefit. VSL#3 may be effective in inducing remission in patients with active UC [5]. These findings suggest that combination of probiotics seems to be more effective in anti-inflammation than single strains. However, the detailed anti-inflammatory mechanism of each probiotic and their interactions are still unclear.

Although some strains of probiotics belonging to the *Lactobacillus* and *Bifidobacterium* genera have been shown to possess anti-inflammatory and immunomodulatory activities, their entire signaling pathways have not been well-documented. There are only limited studies that have examined the inflammation-associated signaling pathways of *L. acidophilus* and *B. animalis* subsp. *lactis*, two common strains of probiotics used in fermented dairy products, especially the comparison and interaction between these probiotics. The aim of the present study was to investigate the anti-inflammatory effects and associated mechanisms of *L. acidophilus*, *B. animalis* subsp. *lactis* and their combination in LPS- and TNF-α-induced inflammatory IECs.

## 2. Materials and Methods

### 2.1. Cell Culture

Human colon cancer cell line HT-29 cells were purchased from the Food Industry Research and Development Institute (Hsinchu, Taiwan) and incubated in 9-cm culture dishes as a monolayer in Roswell Park Memorial Institute (RPMI) 1640 medium supplemented with 10% fetal bovine serum (FBS), 1% L-glutamine (*v*/*v*) and 1% penicillin/streptomycin and maintained at 37 °C in a humidified atmosphere of 5% CO_2_. Cells were sub-cultured following enzymatic digestion using 1% trypsin solution.

### 2.2. Bacterial Strains

*L. acidophilus* and *B. animalis* subsp. *lactis* (Chr. Hansen, Denmark) were grown in the Man-Rogosa-Sharpe (MRS) broth overnight at 37 °C in anaerobic conditions. Viable bacteria were counted by plating serial dilutions on MRS agar. Both strains of probiotics were heat-inactivated by water-bath at 60 °C for 30 min, then collected by centrifugation at 500× *g* for 10 min at 4 °C and washed in phosphate-buffered saline (PBS), as described in previous studies [18,19].

### 2.3. Treatments of Cells

HT-29 cells were seeded in a 24-well microtiter plate at a density of 3 × 10^5^ cells/well in volume of 400 μL medium for one day. Cells as an inflammatory group were treated with non-FBS medium containing LPS (1 μg/mL) and TNF-α (20 ng/mL) from *Escherichia coli* O58:B5 (L6529 Sigma-Aldrich, St. Louis, MO, USA) for 24 h [20]. Cells as intervention groups were added to probiotics at the designated multiplicity of infection (MOI) of 0.1, 1, 10 [21], and incubated in 5% CO_2_ at 37 °C with non-FBS medium containing LPS and TNF-α. The culture medium was harvested and analyzed for IL-8 secretion by enzyme-linked immunosorbent assay (ELISA). We chose the doses of probiotics with an inhibitory effect on IL-8 secretion for comparing the anti-inflammatory effects of single and combined probiotics. Cells were incubated with or without probiotics (*L. acidophilus*, *B. animalis* subsp. *lactis* or both), then medium and cells were collected and analyzed by ELISA and Western blot, respectively.

### 2.4. Cell Viability Assay

HT-29 cells were seeded in a 96-well microtiter plate at a density of 1 × 10^4^ cells/well in volume of 100 μL medium. Cells were treated with *L. acidophilus*, *B. animalis* subsp. *lactis* or both and with LPS and TNF-α for 24 h. After the above treatments, cells were incubated with 3-(4,5-dimethylthiazol-2-yl)-2,5-diphenyltetrazolium bromide (MTT) dye (0.5 μg/μL) at 37 °C for 4 h. Absorbance of the product was read at 570 nm by ELISA reader.

### 2.5. Analysis of IL-8 Secretion by ELISA

IL-8 levels in HT-29 conditioned media were measured by a commercially available ELISA kit (BioLegend, San Diego, CA, USA).

### 2.6. Analysis of Protein Expression by Western Blot

The analytical procedures were modified from previous studies [22,23]. Cells were sonicated for 20 min and then lysed cells were centrifuged at 10,000× *g* for 10 min at 4 °C. Supernatants were collected and the protein concentration was determined using the Bio-Rad Bradford assay (Bio-Rad, Hercules, CA, USA). Samples (each containing 30 μg of supernatant protein) were mixed with NuPAGE LDS sample buffer and β-mercaptoethanol (Invitrogen, Carlsbad, CA, USA), heated at 95 °C for 10 min, cooled to room temperature and electrophoretically resolved on 10% NuPAGE Bis-Tris gels (Invitrogen, Carlsbad, CA, USA). Proteins were then transferred to polyvinylidene difluoride (PVDF) membrane, which was then blocked with blocking buffer (TBST containing 5% albumin) at room temperature for 1 h. The blot was probed with TLR2 (1:1000 dilution, BioVision, Milpitas, CA, USA), phosphorylated p65 NF-κB (*p*-p65 NF-κB, 1:1000 dilution, Bioworld, Dublin, OH, USA), phosphorylated p38 MAPK (*p*-p38 MAPK, 1:1000 dilution, BioVision, Milpitas, CA, USA), cyclooxygenase-2 (COX-2, 1:1000 dilution, Santa Cruz Biotechnology, Santa Cruz, CA, USA), intercellular adhesion molecule-1 (ICAM-1, 1:1000 dilution, BioVision, Milpitas, CA, USA) or vascular cell adhesion molecule-1 (VCAM-1, 1:1000 dilution, Abbiotec, San Diego, CA, USA) polyclonal antibody at 4 °C for 16 h. After being washed extensively to eliminate nonspecific binding, the membrane was incubated with the secondary alkaline phosphatase-labeled antibody at room temperature for 1 h and incubated with 5-bromo-4-chloro-3-indolyl-phosphate/nitroblue tetrazolium (BCIP/NBT) substrate solution (Sigma-Aldrich Inc., St. Louis, MO, USA) for 20 min in the dark. The reactive solution was removed, and the reaction was stopped using 50% methanol. Blot intensities were quantified using Bio-Rad Quantity One software (Bio-Rad Laboratories, Hercules, CA, USA). All experimental data are triplicates.

### 2.7. Statistical Analysis

Data are expressed as means and standard deviations. The difference among groups was analyzed by one-way analysis of variance (ANOVA) and Duncan’s multiple range test using SAS software (SAS Institute, Cary, NC, USA). The significant difference was set at *p* < 0.05.

## 3. Results

### 3.1. Effects of Different Doses of Single Probiotics on IL-8 Secretion in Inflammatory HT-29 Cells

**Figure 1:**
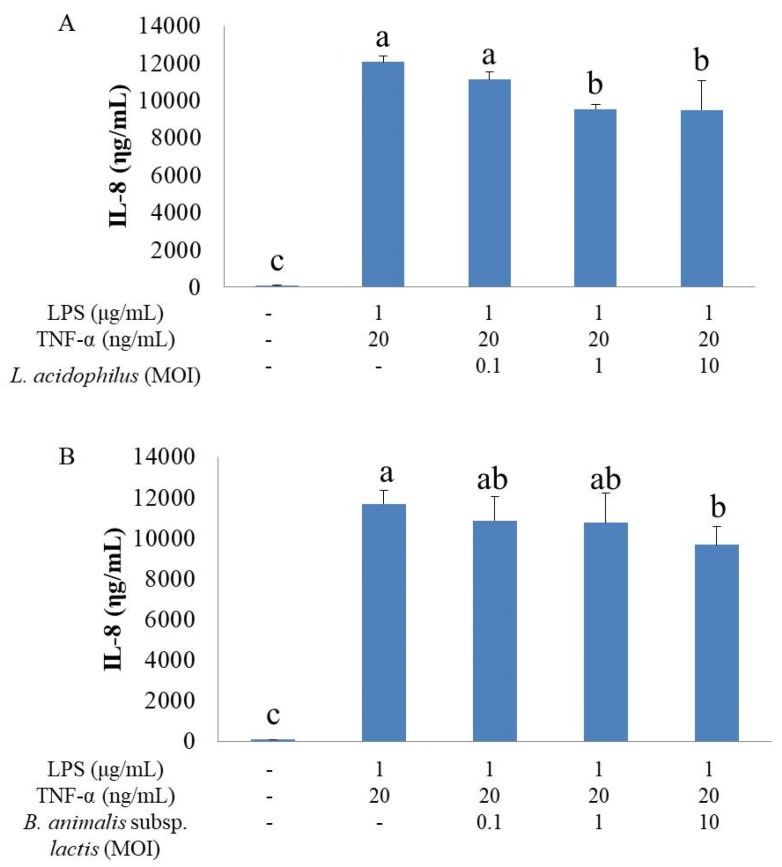
Effects of different doses of single probiotics on IL-8 secretion in inflammatory HT-29 cells. All values except for control are mean ± SD (n = 3). Bars with the same letter are not significantly different from one another determined by Duncan’s multiple range test, *p* < 0.05. (**A**) The inhibitory effect of *L. acidophilus* on IL-8 secretion; (**B**) the inhibitory effect of *B. animalis* subsp. *lactis* on IL-8 secretion.

HT-29 cells stimulated with LPS and TNF-α secreted significantly higher IL-8 levels than did unstimulated cells (Figure 1A,B). *L. acidophilus* at MOI of 1 and 10 significantly decreased IL-8 secretion in inflammatory HT-29 cells (Figure 1A). *B. animalis* subsp. *lactis* at MOI of 10 showed an inhibitory effect on IL-8 secretion (Figure 1B). As a result, we chose *L. acidophilus* at MOI of 1 as group A, *B. animalis* subsp. *lactis* at MOI of 10 as group B, and combination of *L. acidophilus* at MOI of 1 and *B. animalis* subsp. *lactis* at MOI of 10 as group AB for further studies.

### 3.2. Effects of Pro-Inflammatory Factors and Probiotics on Cell Viability

**Figure 2:**
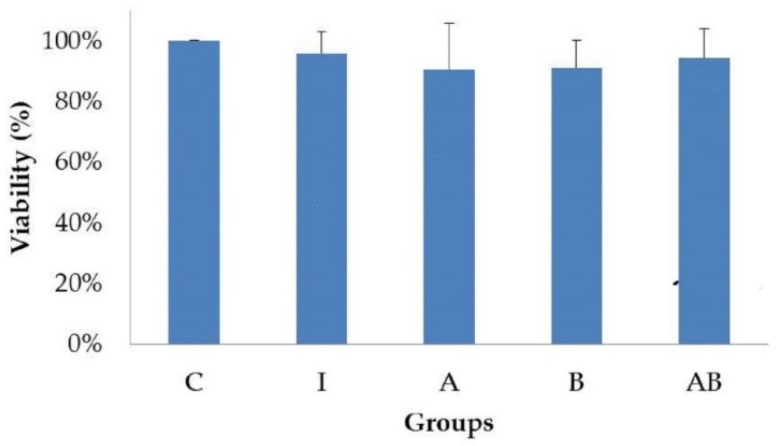
Effects of pro-inflammatory factors and probiotics on viability of HT-29 cells. All values except for control are mean ± SD (n = 3). There is no significant difference among groups determined by Duncan’s multiple range test, *p* < 0.05. C, control; I, TNF-α (20 ng/mL) with LPS (1 μg/mL); A, *L. acidophilus* at MOI of 1 with TNF-α (20 ng/mL) and LPS (1 μg/mL); B, *B. animalis* subsp. *lactis* at MOI of 10 with TNF-α (20 ng/mL) and LPS (1 μg/mL); AB, *L. acidophilus* at MOI of 1 and *B. animalis* subsp. *lactis* at MOI of 10 with TNF-α (20 ng/mL) and LPS (1 μg/mL). Control group is used as the reference.

The viabilities of HT-29 cells were not different among groups after 24 h of incubation (Figure 2), showing that pro-inflammatory factors (LPS and TNF-α) and probiotics (alone or in combination) did not affect the growth of HT-29 cells.

### 3.3. Effects of Probiotics on IL-8 Secretion in Inflammatory HT-29 Cells

**Figure 3:**
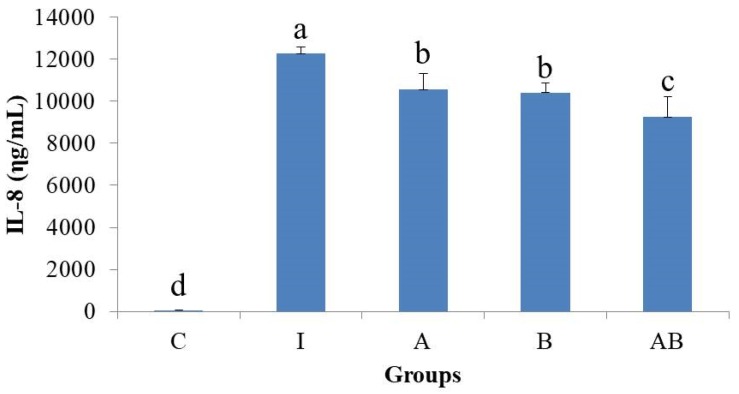
Effects of probiotics on IL-8 secretion in inflammatory HT-29 cells. All values except for control are mean ± SD (n = 3). Bars with the same letter are not significantly different from one another determined by Duncan’s multiple range test, *p* < 0.05. C, control; I, TNF-α (20 ng/mL) with lipopolysaccharide (LPS) (1 μg/mL); A, *L. acidophilus* at MOI of 1 with TNF-α (20 ng/mL) and LPS (1 μg/mL); B, *B. animalis* subsp. *lactis* at MOI of 10 with TNF-α (20 ng/mL) and LPS (1 μg/mL); AB, *L. acidophilus* at MOI of 1 and *B. animalis* subsp. *lactis* at MOI of 10 with TNF-α (20 ng/mL) and LPS (1 μg/mL).

A shown in Figure 3, all probiotics-treated inflammatory HT-29 cells (groups A, B and AB) had significantly lower IL-8 levels than did cells stimulated with LPS and TNF-α (group I). In addition, the IL-8 level was significantly lower in group AB than in either group A or B.

### 3.4. Effects of Probiotics on the Expression of Inflammation-Related Proteins in Inflammatory HT-29 Cells

**Figure 4:**
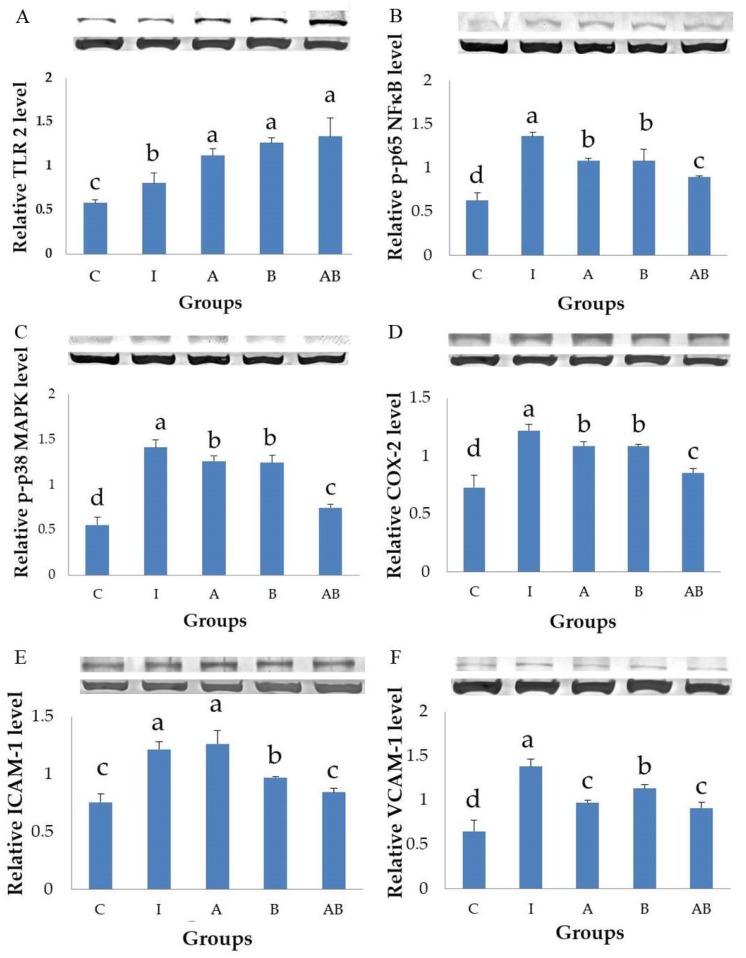
Effects of probiotics on the expression of inflammation-related proteins in inflammatory HT-29 cells. All values are mean ± SD (n = 3). Bars with the same letter are not significantly different from one another determined by Duncan’s multiple range test, *p* < 0.05. C, control; I, TNF-α (20 ng/mL) with LPS (1 μg/mL); A, *L. acidophilus* at MOI of 1 with TNF-α (20 ng/mL) and LPS (1 μg/mL); B, *B. animalis* subsp. *lactis* at MOI of 10 with TNF-α (20 ng/mL) and LPS (1 μg/mL); AB, *L. acidophilus* at MOI of 1 and *B. animalis* subsp. *lactis* at MOI of 10 with TNF-α (20 ng/mL) and LPS (1 μg/mL). (**A**), expression of toll-like receptor 2 (TLR2); (**B**), expression of phosphorylated p65 nuclear factor kappa B (*p*-p65 NFκB); (**C**), expression of phosphorylated p38 mitogen-activated protein kinase (*p*-p38 MAPK); (**D**), expression of cyclooxygenase-2 (COX-2); (**E**), expression of intercellular adhesion molecule-1 (ICAM-1); (**F**), expression of vascular cell adhesion molecule-1 (VCAM-1).

Groups A, B and AB had significantly higher expression of TLR2 (Figure 4A) and lower expressions of *p*-p65 NF-κB (Figure 4B), *p*-p38 MAPK (Figure 4C) and COX-2 (Figure 4D) than did group I. Group AB showed the best inhibitory effect on the expressions of inflammation-related proteins, including *p*-p65 NF-κB (Figure 4B), *p*-p38 MAPK (Figure 4C) and COX-2 (Figure 4D). *B. animalis* subsp. *lactis* treatment (groups B and AB) significantly decreased ICAM-1 expression compared with group I, and this inhibitory effect was more potent in group AB than in group B (Figure 4E). *L. acidophilus* alone (group A) had no inhibitory effect on ICAM-1 expression but it enhanced the efficacy of *B. animalis* subsp. *lactis* when they were used in combination (group AB, Figure 4E). All probiotics-treated groups (groups A, B and AB) had significantly lower VCAM-1 expressions than did group I, while *L. acidophilus* treatment (groups A and AB) showed a better inhibitory effect on VCAM-1 than did *B. animalis* subsp. *lactis* alone (group B), as shown in Figure 4F.

## 4. Discussion

The present study demonstrated that both *L. acidophilus* and *B. animalis* subsp. *lactis* significantly reduced the secretion of IL-8 and the expressions of *p*-p65 NF-κB, *p*-p38 MAPK, VCAM-1 and COX-2, as well as increased the expression of TLR2 in LPS- and TNF-α-induced inflammatory IECs. *B. animalis* subsp. *lactis* significantly reduced the expression of ICAM-1 and this inhibitory effect was enhanced by *L. acidophilus,* which did not affect ICAM-1 expression directly. The combination of two strains showed the best anti-inflammatory activity. These results suggest that both *L. acidophilus* and *B. animalis* subsp. *lactis* possess a potent anti-inflammatory activity in inflammatory IECs and there is an interaction between these two strains of probiotics.

*Lactobacillus* is an important genus of probiotics, which plays an essential role of immunomodulation in the intestinal mucosa. For example, *L. acidophilus* DDS-1 demonstrated a superior survival rate, good adhesion capacity and strong immunomodulatory effect under experimental conditions with or without LPS stimulation [24]. Most *L. acidophilus* species can be well adapted and are able to survive during harsh conditions of digestion [24]. Due to this stability, *L. acidophilus* has been considered an ideal vehicle for mucosal-targeted delivery of drugs [24]. The amount of *L. acidophilus* remained almost unchanged in the gut for a long time [25].

The modulatory effects of *L. acidophilus* on intestinal inflammation and the immune system are dependent on the stimulation in IECs. *L. acidophilus* significantly increased COX-2 expression and prostaglandin E_2_ (PGE_2_) secretion in unstimulated Colo320 cells, but not in TNF-α-induced cells [26]. *L. acidophilus* NCFM could rapidly but transiently induce the production of pro-inflammatory cytokines and chemokines in unstimulated Caco-2 cells [21]. In addition, *L. acidophilus* NCFM activated the pathogen-associated molecular pattern (PAMP) receptor TLR2 and enhanced the phosphorylation of p65 NF-κB and p38 MAPK in unstimulated Caco-2 cells [21]. The activation of TLR2-mediated NF-κB and MAPK signaling pathways played a key role in the production of cytokines and chemokines in IECs [21].

In contrast, *L. acidophilus* showed an anti-inflammatory effect in IECs stimulated by bacterial ligands or pro-inflammatory cytokines. *L. acidophilus* Bar13 were effective in inhibiting IL-8 production in HT-29 cells stimulated by TNF-α, IL-1β or LPS [27]. *L. acidophilus* DDS-1 significantly upregulated anti-inflammatory IL-10 and downregulated pro-inflammatory TNF-α production and IL-8 levels in LPS-treated HT-29 cells [24]. *L. acidophilus* LAB20 increased the transepithelial electrical resistance (TER) of enterocyte monolayers and thus strengthened intestinal barrier function [16]. *L. acidophilus* LAB20 also showed anti-inflammatory capacity by attenuating the LPS-induced IL-8 production in HT-29 cells [16]. The results suggest that this strain may balance the IL-8 expression of IECs in response to stimulation by LPS from Gram-negative bacteria in the intestine. Our study used LPS and TNF-α to induce inflammatory responses of HT-29 cells and the results were in agreement with the above studies, showing an anti-inflammatory effect of *L. acidophilus*. Some animal studies also demonstrated an anti-inflammatory activity of *L. acidophilus* in the intestinal tract. *L. acidophilus* administration reduced colonic leukotriene B_4_ (LTB_4_) production, inducible nitrogen oxide synthase (iNOS) expression and myeloperoxidase (MPO) activity in the TNBS model of rat colitis [17]. *L. acidophilus* inhibited colitis by inducing goblet cell differentiation, interfering with endoplasmic reticulum stress and suppressing NF-κB activation in dextran sulfate sodium (DSS)-treated mice [28]. These findings suggest that *L. acidophilus* can be used as a potent immunomodulator for IBD treatment.

Probiotics modulate immune- and inflammation-associated signaling pathways by gastrointestinal digested products of probiotics and bacterial metabolites. The active compounds of *L. acidophilus* for the anti-inflammatory effect may include either cell components or secretory factors. Cell wall components play a critical role in modulating intestinal inflammation and immune function. Some components of *L. acidophilus*, such as cell wall-associated polysaccharides, proteins and LTAs, could directly inhibit NF-κB activation in HT-29 cells [28]. The surface-layer protein (SLP) from *L. acidophilus* is closely related to a human health-promoting effect via modulation of the immune system. For example, SLP extracted from *L. acidophilus* NCFM was found to inhibit the production of reactive oxygen species (ROS), nitrogen oxide (NO), PGE_2_, TNF-α and IL-1β through attenuating the activation of MAPK and NF-κB in LPS-induced RAW264.7 cells [29]. This SLP inhibited NO and PGE_2_ production by downregulating the protein expression levels of iNOS and COX-2 [29]. On the other hand, the cell-free supernatant of *L. acidophilus* was able to downregulate IL-8 expression in HT-29 cells stimulated by LPS [30]. Otte et al. (2009) indicated that distinct probiotics could specifically and significantly decrease the induced COX-2 expression in IECs, most likely mediated by released factors such as protein, butyrate and propionate and in part by bacterial DNA [26]. A preliminary study of Kainulainen et al. (2015) suggested that exopolysaccharide (EPS) of *L. acidophilus* LAB20 may have a role in its immunomodulatory activity [16].

*Bifidobacteria* make up 3–5% of adult microflora and thus constitute one of the predominant species of the human colonic microflora [31]. They are more abundant in human gut microbiota than *Lactobacilli* [32] and showed a moderate to low adhesion capacity of IECs [24]. *B. animalis* subsp. *lactis* has well-established probiotic characteristics and proven beneficial health effects for gastrointestinal health and immune function [33]. This strain also has potent anti-inflammatory and anti-proliferative effects [10]. The capacity of *Bifidobacteria* to inhibit LPS-induced NF-κB activation is strain-dependent in IECs. For example, *B. bifidum* was reported as a promising candidate for probiotic intervention in inflammatory diseases of the gastrointestinal tract [31]. A recent study screened new probiotic strains for IBD management by combining in vitro and in vivo methods and found that the best anti-inflammatory capacity in peripheral blood mononuclear cells (PBMCs) from healthy donors were observed for *Bifidobacteria*, while the anti-inflammatory profile of *Lactobacilli* was strain-dependent [6]. *B. bifidum* PI22, the strain that exhibited the most protective capacity against acute colitis, was only slightly efficacious against chronic colitis in TNBS-treated mice. This strain inhibited the expression of genes encoding chemokine C-X-C motif ligand 2 (CXCL2), IL-1β, TNF-α, IL-6 and IL-17 [6]. In contrast, *B. lactis* LA804 which was less efficacious in the acute model was the most protective against chronic colitis by downregulating the expression of genes for CXCL2, IL-1β and TNF-α in TNBS-treated mice [6].

Similar to *L. acidophilus*, the modulatory effects of *B. animalis* subsp. *lactis* on intestinal inflammation and the immune system are also dependent on the stimulation used in IECs and animal models. *B. lactis* BB12 transiently triggered the phosphorylation of p65 NF-κB and p38 MAPK in unstimulated native epithelium after the initial monoassociation of rats and in unstimulated Mode-K IECs [34]. TLR2 played an important role in mediating the initial interaction of *B. lactis* BB12 with the intestinal epithelium [34]. This study suggested that probiotics could transiently trigger innate signal transduction and pro-inflammatory gene expression in the intestinal epithelium in the early stages of bacterial colonization [34].

In contrast, *B. animalis* subsp. *lactis* showed an anti-inflammatory effect in IECs stimulated by bacterial ligands or pro-inflammatory cytokines and in animals treated with pro-inflammatory compounds. *B. lactis* inhibited NF-κB and its regulated genes for COX-2, matrix metalloproteinase-9 (MMP-9) and vascular endothelial growth factor (VEGF) in HT-29 cells stimulated with IL-1β, TNF-α or LPS [10]. Another study showed that *B. animalis* ssp. *lactis* UABla-12 was able to downregulate IL-8 levels in LPS-treated HT-29 cells [24]. Our study used LPS and TNF-α to induce inflammatory responses of HT-29 cells and showed an anti-inflammatory effect of *B. animalis* subsp. *lactis*, in agreement with previous studies. Animal studies also confirmed the anti-inflammatory activity of this strain of probiotics. For example, *B. lactis* treatment reduced colonic TNF-α production and the expressions of iNOS and COX-2 in TNBS model of rat colitis [17]. *B. lactis* prevented DSS-induced acute colitis and CAC in mice and could be a therapeutic agent for IBD as well as a potential preventive agent for CAC [10].

Previous studies found that *Bifidobacterium* strains’ DNA was effective in limiting epithelial pro-inflammatory responses in IL-10-deficient mice and in HT-29 cells challenged with TNF-α; however, the pro-inflammatory response to LPS in HT-29 cells was not inhibited by probiotic DNA [31]. These authors indicated that the anti-inflammatory compound is neither actively secreted by *Bifidobacteria* nor released from lysed cells. The mechanisms by which whole cells of *Bifidobacteria* inhibit inflammatory responses are distinct from their DNA [31]. Recent studies have focused on the surface-associated structural components of *Bifidobacteria*, such as EPS, peptidoglycan, LTA and lipoglycans [35]. The biosynthesis of EPS has been described in several species of *Bifdobacterium* and it seems that these molecules play a relevant role in their immunomodulatory and anti-inflammatory capabilities [36].

The synergistic effect of different probiotic strains may have a higher impact on host cells compared to single probiotic strains. Some studies on the anti-inflammatory effects of probiotics were performed using mixtures of different probiotic strains, mainly probiotics belonging to *Lactobacillus* and *Bifidobacterium* genera [31]. Clinical reports have found that administration of mixed probiotics exhibits a combinatorial effect in the host [35]. The enhanced efficacy of multi-strain/combinatorial therapy may arise due to a combination of several probiotic mechanisms, with each strain making their own contributions [35]. Some combined probiotics have been shown to possess a good anti-inflammatory activity in vivo and in vitro. A combination of *Streptococcus thermophilus* ATCC19258 and *L. acidophilus* ATCC4356 had a pronounced effect on the phosphorylation of p38 MAPK and extracellular signal regulated kinase (ERK)1/2 in HT-29 cells with or without the stimulation of interferon-γ (IFN-γ) or TNF-α [30]. In addition, this probiotic combination reduced the phosphorylation of the nuclear factor of kappa light polypeptide gene enhancer in B-cells inhibitor α (IκB-α) [37]. These results suggest that the probiotic combination may activate the MAPK signaling pathway but inhibit the NF-κB signaling pathway in IECs challenged by pro-inflammatory cytokines. VSL#3 probiotics significantly ameliorated the disease activity index (DAI), prevented the decrease of tight junction proteins including occludin and zonula occludens (ZO)-1 and increased the expression of *p*-p38 MAPK and phosphorylated ERK (*p*-ERK) in DSS-treated rats [38]. Furthermore, tight junction proteins (occludin and ZO-1), *p*-p38 MAPK and *p*-ERK in the VSL#3 group were significantly higher than those in either the control or TNF-α group in TNF-α-induced HT-29 cells [38]. This study suggests that VSL#3 probiotics may enhance the tight junction and protect the epithelial barrier in vivo and in vitro by activating the p38 MAPK and ERK signaling pathways. Ultrabletique, a probiotic cocktail, exerted an anti-inflammatory effect and contributed to the rapid recovery of DSS-induced acute colitis in mice [39]. The results were associated with reduced expressions of TLR4, iNOS and NF-κB in colonic tissues [39].

There are limited studies investigating the interaction between different probiotics. *L. acidophilus* Bar13 and *B. longum* Bar33 were effective in inhibiting IL-8 secretion in HT-29 cells stimulated by TNF-α, IL-1β or LPS, and *B. longum* Bar33 presented the highest effect [27]. Both strains showed the potential to protect enterocytes against an acute inflammatory response [27]. However, a combination of these two strains did not show an additive effect. Another study demonstrated that each of *L. acidophilus*, *B. lactis* or their combination had an individual modulatory impact on the immune system, including regulatory T (Treg) cells and effector T helper (Th) cells, of UC patients [13]. However, there is no synergistic effect between these two strains. *B. lactis* had a more stimulatory effect on the Treg cells of UC patients, although it also stimulated the effector Th cells [13]. Based on the IL-10 and transforming growth factor-β (TGF-β) levels of PBMCs stimulated by probiotics, the authors supposed that *B. lactis* could be a better candidate for Treg cells stimulation compared with *L. acidophilus* and mixed probiotics in UC patients. Our study found that both *L. acidophilus* and *B. animalis* subsp. *lactis* significantly suppressed inflammation in LPS- and TNF-α-stimulated HT-29 cells and that the combination of these two strains showed the best anti-inflammatory effect. Our results also indicated that TLR, NF-κB and MAPK signaling pathways and their associated factors, such as adhesion molecules and inflammatory mediators, played important roles in the observed anti-inflammatory activity. The two strains may have an interaction through these pathways.

TLRs are pattern recognition receptors (PRR) expressed by various cells in the gastrointestinal tract, including IECs [8]. TLRs represent the first point of contact between environment and organism and are powerful molecular regulators by which the immune system may sense the environment and protect the host from pathogens or endogenous threats [40]. TLRs are membrane bound and provide pathogen surveillance, which upon ligand binding, activate NF-κB signaling and lead to the production of pro-inflammatory cytokines, chemokines and antimicrobial peptides [12]. TLR signaling in IECs plays several crucial roles in maintaining healthy gut epithelial barrier that includes proliferation of epithelial cells, production of antimicrobials, maintenance of tight junction and modulation of immune system [36]. TLR signaling in the gut is involved in either maintaining intestinal homeostasis or inducing an inflammatory response [8]. Under normal conditions, IECs show low expression of TLRs and are therefore unresponsive to TLR stimuli; under inflammatory conditions, epithelial TLR expression is increased, which contributes to both inflammation and immune tolerance [8]. Epithelial TLR2 activation has been described to protect against barrier disruption in IECs [8].

TLR2 recognizes a wide range of ligands, among which the best characterized are lipoproteins highly expressed on the outer membrane of Gram-positive bacteria [40]. The cell wall of probiotic bacteria contains peptidoglycan, lipoproteins and lipopeptides, described as TLR2 ligands, and thus TLR2 plays an important role in the recognition of anti-inflammatory probiotic bacteria [36]. The immunomodulatory role of LTA in probiotic *Lactobacillus* through TLR2 recognition is well established. Although these biopolymers have long been identified in *Bifidobacterium*, their roles in PRR-mediated interaction in host have not yet been well studied [35]. In contrast, the activation of TLR4 by LPS from Gram-negative bacteria leads to the secretion of pro-inflammatory mediators in the intestinal mucosa, mainly through the activation of the transcription factor NF-κB [39]. TLR2 ligation may induce a suppressive effect on TLR4-mediated inflammatory responses via the expression or activation of TLR negative regulators [12]. The present study showed that *L. acidophilus* and *B. animalis* subsp. *lactis*, either alone or in combination, significantly enhanced TLR2 expression in HT-29 cells stimulated with LPS and TNF-α, suggesting the potential of these probiotics in protecting IECs against bacteria- and cytokine-mediated inflammation.

NF-κB transcriptional system in IEC plays an essential role in the regulation of inflammation in patients with various intestinal disorders [10]. It regulates the transcription of a series of genes involved in acute responses to injury and in chronic intestinal inflammation including the genes for IL-1β, TNF-α, IL-6, IL-8, COX-2, ICAM-1, and so on [31]. NF-κB is activated through a wide range of stimuli such as TNF-α, IL-1β and LPS [31] and acts as a central regulator of the IEC innate immune response and as an essential transcription factor for integrating the pro-inflammatory response to infection with enteroinvasive bacteria [41]. In addition, NF-κB, as an important downstream pathway in LPS-mediated signal responses, is also closely related to tumor growth, inflammation and apoptosis [29]. Probiotics can alter the NF-κB pathway at many different levels including degradation, ubiquitination and inhibition of proteasome function [39]. Induction of the nuclear export of complexes formed by nuclear factor NF-κB and peroxisome proliferator-activated receptor-γ (PPAR-γ) is another anti-inflammatory mechanism induced by probiotics [39].

MAPK proteins, including ERK, c-Jun N-terminal kinase (JNK), and p38 protein kinases, are activated to act on their respective substrates to regulate the inflammatory response in LPS-stimulated cells [29]. Activation of the MAPK signaling pathway by LPS can indirectly activate the downstream NF-κB pathway, initiate protein expression and stimulate complex physiological responses [29]. Probiotics can affect the MAPK signaling pathway independent of NF-κB signaling. Suppression of p38 phosphorylation has been associated with the inhibition of IL-8 secretion without impacting on IL-8 mRNA levels or the activation of NF-κB [14]. Probiotic-induced changes in phosphorylation levels of p38, JNK and ERK1/2 MAPKs can affect cytokine secretion and apoptosis [14].

The present study showed that LPS and TNF-α stimulation significantly increased the phosphorylated forms of p65 NF-κB and p38 MAPK in HT-29 cells, suggesting the activation of NF-κB and MAPK signaling pathways. Inflammatory cells treated with either *L. acidophilus* or *B. animalis* subsp. *lactis* had significantly lower expression of phosphorylated p65 NF-κB and p38 MAPK proteins compared with untreated cells. Furthermore, the combination of *L. acidophilus* and *B. animalis* subsp. *lactis* showed the best inhibitory effect on the activation of NF-κB and MAPK. These results suggest that the two strains of probiotics may exert an anti-inflammatory effect by modulating NF-κB and MAPK signaling pathways in IECs.

IL-8 is a chemokine produced by many types of cells, including IECs [11]. IL-8 is responsible for the initiation of inflammatory cascades and the recruitment of neutrophils into the mucosa of lesions [16]. It also plays important roles in angiogenesis and the metastasis of tumors [11]. In response to enteropathogens infection the IECs release IL-8 and other pro-inflammatory molecules that initiate an acute inflammatory response [27]. A prolonged infection can result in a massive and long-lasting IL-8 release by IECs [27]. LPS, IL-1α, IL-1β, IL-10, IFN-γ and TNF-α regulate IL-8 production in various cell types [11]. HT-29 cells produce IL-8 after stimulation by TNF-α and IL-1β, but not by IFN-γ, IL-10 or IL-13 [11]. TNF-α plays a guiding role in the production of IL-8, and NF-κB establishes a feedback mechanism via autocrine/paracrine factors to regulate the expression and activation of IL-8 [42].

COX-2 plays an important role in inflammation. A variety of extracellular and intracellular stimuli, including LPS and TNF-α, rapidly induce the expression of COX-2 [43]. There are strain- and stimulation-specific effects of probiotics on COX-2 expression in IECs. Both probiotic *E. coli* Nissle and mixed probiotics VSL#3 had no effect on basal COX-2 expression but decreased stimulated COX-2 activity, protein expression and PGE_2_ secretion, while *L. acidophilus* increased COX-2 expression and PGE_2_ secretion in unstimulated Colo320 and SW480 IECs and in gastrin-induced Colo320 cells but not in TNF-α-induced Colo320 cells [26]. Although the precise mechanisms regulating COX-2 expression in IECs have not been fully clarified, activator protein-1 (AP-1) and p38 MAPK are the only regulatory elements identified in IECs so far [26].

The present study found that stimulation by LPS and TNF-α significantly increased the secretory level of IL-8 and the expression of COX-2 in HT-29 cells, showing a typical inflammatory response. In contrast, the inflammatory cells treated with either *L. acidophilus* or *B. animalis* subsp. *lactis* had significantly lower IL-8 level and COX-2 expression compared to those without probiotics treatment. Additionally, combination of *L. acidophilus* and *B. animalis* subsp. *lactis* showed the best inhibitory effect on IL-8 secretion and COX-2 expression. These effects are in agreement with those on NF-κB and MAPK and suggest that these two strains of probiotics are able to suppress inflammation by modulating IL-8 secretion and COX-2 expression in IECs.

Cell adhesion molecules (CAMs) are categorized as cadherins, selectins, integrins and immunoglobulin-like adhesion molecules (IgCAMs) such as mucosal addressin cell adhesion molecule 1 (MAdCAM-1), ICAM-1 and VCAM-1. They are required for the binding of leukocytes to tissue components and the directional movement of leukocytes toward inflamed sites [44]. Human IECs are normally lack of ICAM-1 and VCAM-1 expressions but induction of their expressions has been reported in response to pro-inflammatory cytokines such as IL-1β, IL-6 and IFN-γ [45]. Our study also induced the expressions of ICAM-1 and VCAM-1 by LPS and TNF-α. ICAM-1 and VCAM-1 are closely related to NF-κB and MAPK signaling pathways. The binding sites for NF-κB have been identified in the promoter regions of the genes for ICAM-1 and VCAM-1 [46]. Another study has indicated that there is a possible relationship between inhibition of MAPKs and decreased expression of ICAM-1 and VCAM-1 [45].

ICAM-1 is constitutively expressed by several types of cells, including leukocytes, endothelial cells and IECs [47,48]. The major agonists for ICAM-1 expression are bacterial products (such as LPS) and pro-inflammatory cytokines (such as TNF-α, IL-1 and IFN-γ) released at inflammatory sites [47]. Expression of ICAM-1 in human IECs is upregulated following infection with invasive bacteria and stimulation with TNF-α and IFN-γ [47]. Transcription of ICAM-1 is under regulation by cytokines such as IL-1β, TNF-α and IFN-γ as well as by growth factors and stress oxidants through various signaling pathways, including NF-κB and AP-1 pathways [44]. ICAM-1 plays an important role in both innate and adaptive immune responses. It is involved in the transendothelial migration of leukocytes to inflammatory sites, as well as interactions between antigen presenting cells (APC) and T cells [48]. ICAM-1 on IECs is involved in the immune defense, including attraction of neutrophils to the surface defect, adhesion of transmigrated neutrophils to face the luminal content and binding of microbial surface molecules [44]. Both active UC and CD are associated with marked infiltration of inflammatory cells, while ICAM-1 is involved in leukocyte infiltration [49]. ICAM-1 plays a specific role in the pathogenesis of IBD, so loss of ICAM-1 function may be effective in controlling IBD [44].

VCAM-1 is inducible and predominantly expressed in endothelial cells and also on the surface of other cells, including macrophages and cancer cells [50]. The expression of VCAM-1 is activated by pro-inflammatory cytokines, including TNF-α, and also by ROS, oxidized low-density lipoprotein (oLDL), high glucose concentration, TLR agonists (such as LPS) and shear stress [50]. VCAM-1 is an important modulator of lymphocyte and monocyte trafficking [46]. VCAM-1 was originally identified as a cell adhesion molecule that helps regulate inflammation-associated vascular adhesion and the transendothelial migration of leukocytes such as macrophages and T cells [50]. Recent evidence suggests that VCAM-1 is closely associated with the progression of various immunological disorders and cancers, so it is a potential therapeutic target in these diseases [50]. Experimental studies have shown that blockade of VCAM-1 results in a higher inhibition of leukocyte adhesion in colonic venules and a potent effect on preventing or reversing established inflammation in various animal models [51,52,53].

In human intestinal microvascular endothelial cells, ICAM-1 is constitutively expressed and VCAM-1 is not detectable in basal conditions but a marked increase in both adhesion molecules is observed after challenges of these cells with IL-1β, TNF-α or LPS [46]. In the murine intestine, the constitutive level of VCAM-1 expression is substantially lower than that of ICAM-1; however, a significant increase in the endothelial cell surface density of VCAM-1 is noted by cytokine stimulation [46]. Remarkable increases in VCAM-1 expression have also been documented in rodent models of chemically induced colitis, as well as in colitis of IL-10 knockout mice [46,51,52]. In these animal models, the expression of ICAM-1 was not increased or was only slightly elevated, whereas increased expressions of MAdCAM-1 and VCAM-1 were observed. VCAM-1, in contrast with ICAM-1, is not involved in physiological leukocyte recirculation and selective blockade of its function might attenuate the inflammatory response without altering physiological immune mechanisms [46]. Specific blockade of the adhesion molecules such as ICAM-1 involved in leukocyte recruitment to the inflamed intestine is considered to be one of the most promising therapeutic targets for IBD [53,54].

Limited studies have investigated the effect of probiotics on adhesion molecules. Riedel et al. (2006) reported that *B. bifidum* S17 decreased mRNA level of ICAM-1 in LPS-induced HT-29 cells [31]. Angulo et al. (2006) demonstrated that *L. casei* attenuated leukocyte recruitment in experimental colitis induced by TNBS in rats [55]. This effect was possibly related to the suppression of ICAM-1 upregulation, although VCAM-1 expression was unaffected. Chu et al. (2010) indicated that *L. plantarum* treatment improved the histological damage score and interfered with the upregulation of adhesion molecules such as ICAM-1 in IL-10 knockout mice [49]. In addition to whole probiotics, the cell wall components of probiotics also play an important role in regulation of adhesion molecules and other inflammatory-related factors. Kim et al. (2012) showed that LTA from *L. plantarum* inhibited TNF-α-induced inflammatory responses, including increased IL-8, iNOS, NO and ICAM-1, in HT-29 cells through inhibition of the NF-κB and MAPK signaling pathways [11].

Our study successfully induced strong expressions of ICAM-1 and VCAM-1 in HT-29 cells stimulated with LPS and TNF-α. Interestingly, *L. acidophilus* and *B. animalis* subsp. *lactis* showed a different impact on ICAM-1 and VCAM-1. *L. acidophilus* and *B. animalis* subsp. *lactis*, either alone or in combination, significantly decreased VCAM-1 expression in inflammatory HT-29 cells compared with those untreated with probiotics. *L. acidophilus* showed higher efficacy on VCAM-1 inhibition than did *B. animalis* subsp. *lactis*. However, *L. acidophilus* alone did not inhibit ICAM-1 expression but enhanced the efficacy of *B. animalis* subsp. *lactis* which significantly decreased ICAM-1 expression in inflammatory HT-29 cells. It shows a synergistic effect between two strains of probiotics. ICAM-1, associated with the recruitment and adhesion of neutrophils, is known to play a determining role in acute inflammatory conditions, while VCAM-1 is more relevant as a mediator of chronic inflammatory diseases in which the recruitment of lymphocytes is the main cellular event [44,55]. Blocking ICAM-1 and VCAM-1 may have therapeutic benefits for the acute inflammatory component of IBD in SAMP-1/Yi adoptive transfer model mice [56]. These findings suggest that *B. animalis* subsp. *lactis* may have the potential to alleviate acute inflammation in the gut, while *L. acidophilus* may be a candidate for remission of chronic intestinal inflammation. Combination of two strains seems to have the best anti-inflammatory activity in the intestinal tract and may be a candidate adjuvant for IBD treatment.

## 5. Conclusions

In conclusion, the present study showed that *L. acidophilus* and *B. animalis* subsp. *lactis* exerted a potent anti-inflammatory effect through modulating TLR2-mediated NF-κB and MAPK signaling pathways in inflammatory IECs. Both strains, especially their combination, may have the potential to be developed as a novel adjuvant for IBD therapy.
